# Conduit Artery Diameter During Exercise Is Enhanced After Local, but Not Remote, Ischemic Preconditioning

**DOI:** 10.3389/fphys.2018.00435

**Published:** 2018-04-24

**Authors:** Scott Cocking, N. T. Cable, Mathew G. Wilson, Daniel J. Green, Dick H. J. Thijssen, Helen Jones

**Affiliations:** ^1^Athlete Health and Performance Research Centre, Aspetar Orthopaedic and Sports Medicine Hospital, Doha, Qatar; ^2^Research Institute for Sport and Exercise Science, Liverpool John Moores University, Liverpool, United Kingdom; ^3^Department of Sport Science, Aspire Academy, Doha, Qatar; ^4^School of Sport, Exercise and Rehabilitation Sciences, University of Birmingham, Birmingham, United Kingdom; ^5^Sport and Exercise Science, School of Human Sciences, Faculty of Science, The University of Western Australia, Crawley, WA, Australia; ^6^Department of Physiology, Radboud University Medical Center, Radboud Institute for Health Sciences, Nijmegen, Netherlands

**Keywords:** ischemic preconditioning, cardiovascular, endothelial function, handgrip exercise, blood flow

## Abstract

**Introduction:** The ability of ischemic preconditioning (IPC) to enhance exercise capacity may be mediated through altering exercise-induced blood flow and/or vascular function. This study investigated the hypothesis that (local) IPC enhances exercise-induced blood flow responses and prevents decreases in vascular function following exercise.

**Methods:** Eighteen healthy, recreationally trained, male participants (mean ±*SD*: age 32 ± 8 years; BMI 24.2 ± 2.3; blood pressure 122 ± 10/72 ± 8 mmHg; resting HR 58 ± 9 beats min^-1^) received IPC (220 mmHg; 4 × 5-min bilateral arms), REMOTE IPC (220 mmHg; 4 × 5-min bilateral legs), or SHAM (20 mmHg; 4 × 5-min bilateral arms) in a counterbalanced order prior to 30-min of submaximal (25% maximal voluntary contraction) unilateral rhythmic handgrip exercise. Brachial artery diameter and blood flow were assessed every 5-min throughout the 30-min submaximal exercise using high resolution ultrasonography. Pre- and post-exercise vascular function was measured using flow-mediated dilation (FMD).

**Results:** IPC resulted in enlarged brachial artery diameter during exercise [0.016 cm (0.003–0.03 cm), *P =* 0.015] compared to REMOTE IPC, but blood flow during exercise was similar between conditions (*P* > 0.05). Blood flow (l/min) increased throughout exercise (time: *P <* 0.005), but there was no main effect of condition (*P =* 0.29) or condition ^∗^ time interaction (*P =* 0.83). Post-exercise FMD was similar between conditions (*P* > 0.05).

**Conclusion:** Our data show that local (but not remote) IPC, performed as a strategy prior to exercise, enhanced exercise-induced conduit artery diameter dilation, but these changes do not translate into increased blood flow during exercise nor impact post-exercise vascular function.

## Introduction

Ischemic preconditioning (IPC) is an intervention whereby three to four brief periods of ischemia, followed by tissue reperfusion, confer protection against subsequent ischemic insults (Murry et al., 1986). A single episode of IPC applied to both the exercising (IPC) and non-exercising limbs (REMOTE IPC) can enhance exercise performance (Bailey et al., 2012b; Barbosa et al., 2015), although the ability of IPC to positively impact performance based on the findings of a meta-analysis remains to be determined (Marocolo et al., 2015). To date, the mechanism underlying any ergogenic response remains speculative. It is hypothesized that IPC may induce alterations in skeletal muscle, via mitochondrial activation (Kido et al., 2015), and better maintenance of vascular function following exercise (Bailey et al., 2012a). Blood flow to exercising muscles is regarded as an important factor in determining the muscle’s capacity to generate and perform muscle work (Joyner and Casey, 2014). An intervention capable of enhancing oxygen delivery and nutrient exchange within the working muscles could be vital for enhanced exercise performance. Additionally, the suggestion that (REMOTE)IPC can enhance high-intensity muscular performance, via delaying resistance to fatigue development in small muscle mass exercise (Barbosa et al., 2015) and augment in maximal-intensity larger muscle performance outcome (Kraus et al., 2015; Paradis-Deschênes et al., 2016) remains an interesting area of research. Regardless of performance outcome in these tasks, the mechanisms underpinning any potential performance changes currently remain elusive.

In an isolated exercise model (handgrip exercise), one previous study demonstrated that handgrip performance (time to exhaustion) was enhanced after REMOTE IPC, yet no change in blood flow occurred (Barbosa et al., 2015). The duration of handgrip exercise was likely too short to assess a steady-state exercise response on the vasculature. Nonetheless, some evidence suggests that REMOTE IPC can affect the dilator responses in the contralateral brachial artery (Enko et al., 2011). For example, Bailey et al. (2012a,b) found that REMOTE IPC negated the usual post-exercise reductions in brachial artery endothelial function, measured via the flow-mediated dilation (FMD) technique, after a 5-km time trial on a treadmill (Bailey et al., 2012a). A subsequent study by Horiuchi et al. (2015) used near-infrared spectroscopy (NIRS) to measure local limb oxygenation as an index of forearm blood flow at rest, light- [10% maximal voluntary contraction (MVC)], and moderate-intensity (25% MVC) handgrip exercise. They found a larger vasodilation during moderate-intensity exercise when the bout was preceded by IPC (Horiuchi et al., 2015). Taken together, there is accumulating evidence that IPC and REMOTE IPC have a direct role on the vasculature which could contribute to the ergogenic effects during exercise. However, no study has explored whether IPC could directly affect the exercise-induced changes in diameter, blood flow, and function, and whether these changes are different between IPC and REMOTE IPC, while using a SHAM condition to assess control responses.

The primary aim of this study was to examine the exercise-mediated changes in artery diameter and blood flow in response to IPC and REMOTE IPC in healthy individuals. We hypothesized that both IPC and REMOTE IPC would cause a diameter increase, accommodating a larger blood flow during exercise. A secondary aim of the study was to examine whether IPC and REMOTE IPC could prevent the usual decline in post-exercise vascular function compared to a SHAM condition. Finally, we examined whether (REMOTE)IPC is capable of enhancing MVC capacity when preceded by 30 min of submaximal exercise.

## Materials and Methods

### Participants

Eighteen healthy, recreationally trained males (mean ± SD: age 32 ± 8 years; BMI 24.2 ± 2.3; blood pressure 122 ± 10/72 ± 8 mmHg; resting HR 58 ± 9 beats min^-1^) were recruited and provided written informed consent in accordance with the Declaration of Helsinki. Physical Activity Readiness Questionnaires were administered to ensure no participant had any cardiovascular or metabolic health issues that would prevent participation. Participants refrained from exercise, and consumption of alcohol at least 24 h and caffeine 6 h prior to all laboratory visits. Participants were instructed to standardized food and drink prior to each trial and visited the laboratory at the same time (±1 h) for each trial to delimit the influence of circadian variation on outcome measures (Jones et al., 2010). Laboratory visits took place in a temperate environment (dry bulb temperature 22.9 ± 0.6°C; relative humidity 49.6 ± 6.7%; barometric pressure 758.4 ± 2.1 mmHg). The study was approved by the local Ethics Committee (Anti-Doping Lab Qatar, Doha, IRB F2016000128).

### Experimental Design

**FIGURE 1:**
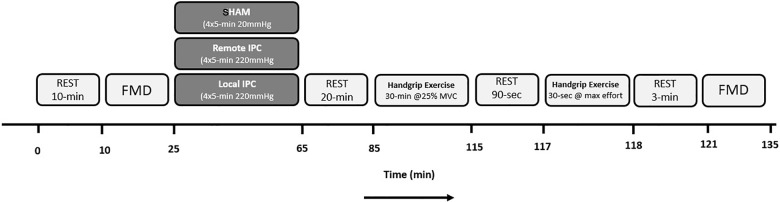
Protocol schematic detailing experimental laboratory visits.

Participants reported to the laboratory on three separate occasions, performing unilateral handgrip exercise that was preceded by either Local (IPC), Remote (REMOTE IPC), or SHAM condition (in a randomized order). Upon arrival, a resting FMD test was performed followed by either IPC, REMOTE IPC, or SHAM. Both IPC (upper arms) and REMOTE IPC (upper legs) consisted of four sets of 5 min cuff inflation of the limbs (220 mmHg) followed by 5 min reperfusion periods. SHAM consisted of four sets of 5 min cuff inflations at low pressure (20 mmHg) on the upper legs. Following a 20 min rest period, participants performed 30 min of rhythmic (30 contraction/relaxation cycles/min), submaximal handgrip exercise at 25% MVC. Brachial artery blood flow was monitored throughout the exercise bout. Participants rested for 90 s and then performed a 30 s “all out” MVC handgrip contraction. A post-exercise FMD test was then performed 3 min after the completion of the maximal contraction bout (**Figure 1**). Visits were separated by 4–7 days.

### Assessing Maximum Voluntary Contraction

Participants attended the laboratory on their initial visit and performed short (3 s) maximal voluntary handgrip isometric contractions (MVC); with each effort separated by 90 s rest. Each participant produced three efforts in total. A dynamometric handheld force transducer using MP35 hardware (Biopac Systems, Santa Barbara, CA, United States) and dedicated software (BSL Pro Version 3.6.7, Biopac Systems, Santa Barbara, CA, United States) was used to determine force generation. The maximum recorded value (kg) from these three efforts was used to determine MVC. For MVC determination, the signal was amplified (gain = 200) and recorded at a sampling frequency of 10 kHz. A standardized load of 20 kg (20 kg weight plate) was placed on the transducer 10 min after the system was turned on in order to calibrate the system prior to each trial.

### Brachial Artery Endothelial Function

Following 5 min of supine rest, the participants arm was extended and positioned at an angle of approximately 80° from the torso. A cuff attached to a rapid inflator was placed distal to the olecranon process on the forearm. A 15 MHz multi-frequency linear array probe attached to a high-resolution ultrasound machine (T3000: Terason, Burlington, MA, United States) was used to image the brachial artery in the distal third of the upper arm. Once a suitable image was obtained, the probe was held in position and settings were altered to optimize the longitudinal B-mode image of the lumen–arterial wall interface. Settings were identical between all FMD assessments. When collecting continuous Doppler velocity, the lowest insonation angle (<60°) was used. Following 1 min of baseline recording, the forearm cuff was inflated (>200 mmHg) for 5 min. Image recording commenced 30 s before cuff deflation and continued for 3 min thereafter (Black et al., 2008). Variables analyzed from FMD analysis that are displayed in **Table 3** consist of: peak diameter response (defined as the maximum brachial artery diameter obtained post-cuff release); time to peak diameter (defined as the time from cuff release to peak diameter response), and shear rate (four times velocity divided by diameter) area under the curve (SR_AUC_; defined as the measured shear from the point of cuff release to peak dilation).

### IPC Protocol

Ischemic preconditioning was performed in the supine position and cuff inflation pressure set at a standardized pressure (220 mmHg) in all experimental IPC conditions. With the use of a rapid inflator (E20) and air source (AG101) (Hokanson, Bellevue, WA, United States), 13.5 cm wide cuffs were inflated to 220 mmHg for 5 min, with the aim of preventing arterial inflow (Sharma et al., 2014). Subsequently, cuffs were deflated for 5 min, allowing reperfusion. This cycle was repeated four times in both IPC and REMOTE IPC conditions. A SHAM condition was also performed, in which cuffs were placed bilaterally on the upper thighs (4 × 5 min) and inflated to 20 mmHg. In each experimental trial, participants gave a perceived-discomfort rating following each IPC cycle. The discomfort rating was established using a numerical rating scale ranging from 0 (no discomfort) to 10 (maximum discomfort) (Ferreira-Valente et al., 2011) and is included for descriptive purposes (**Table 4**). A 20-min rest period was undertaken prior to experimental handgrip exercise performance. No participants were informed about the purpose or hypothesis of the study.

### Handgrip Exercise

For all experimental trials, handgrip exercise was set at a submaximal intensity of 25% MVC. All sessions were performed at the same time of day, relative to the MVC visit in order to limit time of day effect on grip strength variation (Jasper et al., 2009). Participants remained in the supine position, with the dominant arm placed at an angle approximately 80° from the torso. Participants performed 30-min of rhythmic handgrip contractions on a dynamometric handheld force transducer using MP35 hardware (see the section “Assessing Maximum Voluntary Contraction” for details). The force scale was adjusted accordingly and the 25% MVC target intensity was clearly displayed on a screen in front of the participant. Real-time force (kg) feedback allowed participants to ensure they were working at the correct intensity for the duration of the trial. The rate of contractions was dictated by a metronome set at a rate of 30 contraction–relaxation cycles/minute.

### Maximal Voluntary Contraction Task

Following cessation of rhythmic handgrip exercise, a 90-s recovery was allocated. Once rest time had elapsed, participants completed a 30-s forearm MVC using the same dynamometric handheld force transducer previously mentioned. Both peak force (kg) and area under the curve (kg) were recorded.

### Blood Flow Measurements

During the handgrip task, brachial artery diameter and blood velocity were measured with a linear array probe attached to a high-resolution ultrasound machine (T3000: Terason, Burlington, MA, United States). The probe was placed on the distal third of the upper arm for image consistency. Blood flow velocity (derived from Doppler region of interest at 30 Hz) was measured with an insonation angle <60°. Measurements were taken for 60 s per time point and mean values were calculated. Measures were performed at 1, 5, 10, 15, 20, 25, and 29 min during the 30-min handgrip task. Antegrade blood velocity was measured during the interval between contractions, while retrograde blood velocity occurred during muscular contraction.

### Brachial Artery Diameter and Blood Flow Analysis

Custom-designed edge-detection software was used to analyze all recordings. This method allowed analysis to be performed largely independent of investigator bias. Blood flow (the product of lumen cross-sectional area and Doppler velocity) was determined from the synchronized diameter and velocity data at a sampling rate of 30 Hz. Shear rate (s^-1^) (independent of viscosity) was calculated as four times mean blood velocity/vessel diameter. The semi-automated software, compared with manual methods, significantly reduces observer error and has shown previous intra-observer coefficients of variation (CoV) of 6.7% (Woodman et al., 2001). Intra-observer CoV in measurement of baseline arterial diameter in the current study was reported as 2.2 ± 1.4%. All files were analyzed by the same member of the research team (SC) to maximize reliability between trials. We also controlled for the baseline diameter measured before the introduction of hyperemia in each FMD test. This allometric approach is more accurate for scaling changes in diameter than simple percentage change, which makes implicit assumptions about the relationship between baseline diameter and peak diameter (Packard and Boardman, 2008; Atkinson and Batterham, 2013).

### Statistical Analysis

All data were analyzed using linear mixed modeling. The primary outcome variable was blood flow response during exercise. This and all variables measured during exercise were analyzed with condition (three levels: IPC, REMOTE IPC, and SHAM) and time point (seven levels: minutes 1, 5, 10, 15, 20, 25, and 29 of exercise). Brachial artery endothelial function measurements were analyzed with condition (three levels: IPC, REMOTE IPC, and SHAM) and time-point (two levels: pre- and post-exercise). The least-significant method was employed for pairwise comparisons (Perneger, 1998). Data are presented in the text as the mean difference (95% CI). The level of significance (alpha) was set at *P* = 0.05. Any *P-*value that was reported as 0.00 in SPSS is reported in the current manuscript as *P* < 0.005.

## Results

### Artery Responses During Exercise

#### Diameter

**FIGURE 2:**
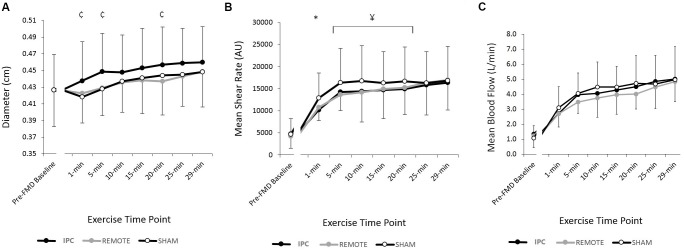
Mean ± SD: data reported for **(A)** brachial artery diameter (cm) **(B)** shear rate (s^-1^), and **(C)** blood flow (l/min) during 25% MVC handgrip exercise for each conditions. 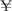 represents a significant difference between IPC and SHAM; 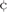 represents a significant difference between IPC and REMOTE; and ^∗^ represents a significant difference between IPC and both conditions.

Diameter increased throughout the 30-min exercise bout (*P <* 0.005) (**Figure 2**). There was a main effect of condition (*P* = 0.03) whereby diameter during exercise following IPC was 0.016 cm (0.003–0.03) greater compared to REMOTE IPC (*P =* 0.015), while the difference with SHAM did not reach statistical significance [0.013 (-0.005 to 0.03) cm; *P =* 0.16]. Diameter changes between REMOTE IPC and SHAM [-0.03 (-0.16 to 0.10) cm] were not different (*P* = 0.66). There was no condition ^∗^ time interaction effect (*P =* 0.48).

#### Blood Velocity

There was a main effect of time (*P <* 0.005) and condition (*P <* 0.005) with both IPC [-2.6 (-3.9 to -1.4) cm/s; *P <* 0.005] and REMOTE IPC [-2.2 (-3.4 to -1) cm/s; *P =* 0.001] resulting in lower velocity *versus* SHAM. There was no difference between IPC and REMOTE IPC [-0.42 (-1.95 to 1.12) cm/s; *P* = 0.89]. There was no condition ^∗^ time interaction (*P =* 0.78) (**Table 1**).

**Table 1:** Mean ± SD reported for blood velocity (cm/s), antegrade shear (SR_ANT_), and retrograde shear (SR_RET_) for each time point during submaximal rhythmic handgrip exercise.

Variable	Condition	Time point								*P-*value
		Pre-exercise	1-min	5-min	10-min	15-min	20-min	25-min	29-min	condition
Velocity (cm/s)	IPC	9.21 ± 5.33	18.2 ± 5.8¥	25.8 ± 10	26.3 ± 9.1¥	27.1 ± 9	27.8 ± 9.4	29.7 ± 9.7	30.7 ± 13	
	REMOTE	8.28 ± 5.23	18.9 ± 5.2	24.1 ± 5.6#	25.3 ± 10.5	26.7 ± 10.7	27.1 ± 8.8	29.6 ± 11.6	31 ± 10.3	*P <* 0.005
	SHAM	7.93 ± 6.71	22.2 ± 6.7	28.5 ± 11.7	30 ± 12.5	29.5 ± 11.1	30.5 ± 12.7	29.8 ± 11.9	31.1 ± 13.2	
Antegrade	IPC	5323 ± 2390	11,440 ± 4188*	15,962 ± 7200¥	16,089 ± 6113¥	16,244 ± 6367¥	16,478 ± 6248¥	17,122 ± 6530	17,608 ± 8131	
shear (SR_ANT_)	REMOTE	4850 ± 2466	12,382 ± 2860¢	15,576 ± 3980	16,183 ± 6746	16,674 ± 6695	16,991 ± 6053	17,931 ± 7314	18,466 ± 6627	*P <* 0.005
	SHAM	4861 ± 3812	14,419 ± 6164	18,333 ± 8191	18,620 ± 8225	18,298 ± 7368	18,519 ± 7970	18,172 ± 7248	18,493 ± 7932	
Retrograde	IPC	(-)588 ± ± 692	(-)1250 ± 709	(-)1718 ± 900	(-)1685 ± 853	(-)1535 ± 765	(-)1564 ± 834	(-)1315 ± 812¥	(-)1236 ± 787	
shear (SR_RET_)	REMOTE	(-)619 ± 624	(-)1633 ± 1020	(-)1983 ± 1171	(-)2036 ± 981	(-)1765 ± 962	(-)1771 ± 852	(-)1624 ± 885	(-)1536 ± 795	*P =* 0.27
	SHAM	(-)651 ± 695	(-)1563 ± 919	(-)2021 ± 1109	(-)1911 ± 1126	(-)2004 ± 1251	(-)1859 ± 1148	(-)1919 ± 1089	(-)1711 ± 1034	


*#represents a significant difference between REMOTE and SHAM; ¥represents a significant difference between IPC and SHAM; aaarepresents a significant difference between IPC and REMOTE; and ^∗^represents a significant difference between IPC and both conditions.*

#### Blood Flow

Blood flow increased throughout exercise (time: *P <* 0.005), but there was no main effect of condition (*P =* 0.29) or condition ^∗^ time interaction (*P =* 0.83) (**Figure 2**).

#### Shear Rate, Antegrade Shear, and Retrograde Shear

There was a main effect of time for mean shear rate (SR_MEAN_) (**Figure 2**), antegrade shear (SR_ANT_), and retrograde shear (SR_RET_) (**Table 1**), with all three variables increasing with exercise duration (all *P <* 0.005, respectively). A main effect of condition was present for SR_MEAN_ (*P <* 0.005) with IPC being lower [-688 s^-1^ (-1369 to -6); *P =* 0.048] than REMOTE IPC and lower [-1756 s^-1^ (-2435 to -1078); *P <* 0.005] than SHAM. SR_MEAN_ for REMOTE IPC was -1069 s^-1^ (-1736 to -402) lower than SHAM (*P =* 0.002). There was no condition ^∗^ time interaction (*P =* 0.76).

There was a significant effect of condition (*P <* 0.005) for SR_ANT_ (**Table 1**), with IPC resulting in -938 s^-1^ (-1648 to -227; *P =* 0.01) lower SR_ANT_ compared to REMOTE IPC and -2116 s^-1^ (-2823 to -1408; *P* < 0.005) lower SR_ANT_ compared to SHAM. REMOTE IPC resulted in -1178 s^-1^ (-1873 to -483; *P =* 0.001) lower SR_ANT_ when compared to SHAM. There was no condition ^∗^ time interaction (*P =* 0.89). Additionally, no main effect of condition or condition ^∗^ time interaction was evident in SR_RET_ (*P =* 0.68).

### Brachial Artery Endothelial Function

**FIGURE 3:**
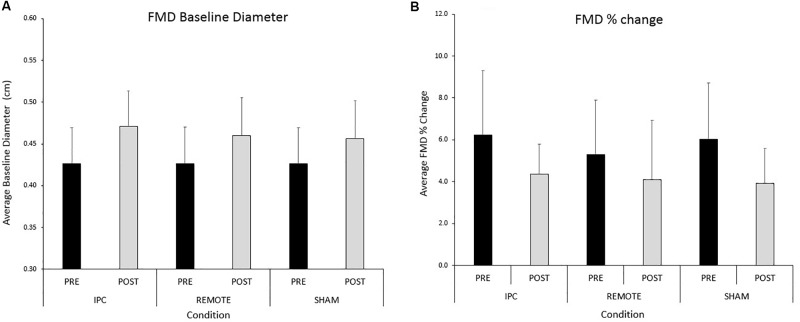
Mean ± SD: data reported for **(A)** baseline diameter and **(B)** FMD pre- and post-exercise for each condition.

A significant main effect of time was evident for brachial artery FMD (**Figure 3**). FMD decreased by 1.9% (-2.8, -1.03) from pre- to post-exercise (*P <* 0.005). There were no main effects of condition or condition ^∗^ time interaction (*P* > 0.05, respectively). Results did not alter when the data were allometrically scaled for baseline diameter.

A significant main effect of time (*P <* 0.005) was evident for peak diameter (**Table 2**). Peak diameter increased by 0.03 (0.024–0.036) cm from pre- to post-exercise. There was evidence of a main effect of condition but this did not reach statistical significance (*P =* 0.09). Peak diameter was 0.008 (0.001–0.16) cm larger after IPC compared to SHAM (*P =* 0.03). There were negligible differences in peak diameter between IPC and REMOTE IPC [0.005 (-0.003 to 0.012) cm; *P =* 0.21] and also between REMOTE IPC and SHAM conditions [0.004 (-0.004 to 0.011) cm; *P =* 0.35]. There was no interaction between condition ^∗^ time (*P* = 0.14).

**Table 2:** Pre- and post-exercise FMD responses (mean ± SD) for peak diameter (cm), time to peak dilation (s), and baseline shear rate (SR_AUC_).

Condition	IPC		REMOTE		SHAM		*P*-Value
Time point	Pre	Post	Pre	Post	Pre	Post	Condition
Peak diameter response (cm)	0.45 ± 0.05	0.49 ± 0.05 ¥	0.45 ± 0.04	0.48 ± 0.04	0.45 ± 0.05	0.47 ± 0.05	*P =* 0.09
Time to peak (s)	65 ± 37	108 ± 42	62 ± 21	100 ± 33	78 ± 40	99 ± 31	*P =* 0.71
Response SR_AUC_	19,038 ± 8724	42,642 ± 16585	16,678 ± 6983	36,176 ± 10,079	21,093 ± 8114	39,001 ± 12,777	*P =* 0.18


*¥ represents a significant difference between IPC and SHAM.*

A main effect of time was evident for baseline diameter (cm) (**Figure 3**), time to peak diameter (seconds), and shear rate under-the-curve (from cuff deflation to peak dilation; SR_AUC_) (**Table 2**) which all increased from pre- to post-exercise (*P* < 0.005; **Table 3**). There was no main effect of condition or condition ^∗^ time interaction for any variable (*P* > 0.05, respectively).

**Table 3:** Mean ± SD values for 30 s mean force production (kg) and peak (1 s) force production (kg), respectively, across conditions.

Condition	LOCAL	REMOTE	SHAM	*P-*Value
30 s force (kg)	20.9 ± 6.3	22.1 ± 6.2	21.5 ± 4.9	0.4
Peak force (kg)	25.3 ± 6.3	25.6 ± 6.7	25.6 ± 5.6	0.91


**Table 4:** Mean ± SD values for perceived discomfort of ischemic preconditioning (IPC) and SHAM interventions.

	Perceived discomfort of condition (ratings 0–10)					
	Average	0–10 min	10–20 min	20–30 min	30–40 min	Mean discomfort rating
IPC	3.4 ± 0.3	3.8 ± 2	3.4 ± 1.9	3.3 ± 2.1	3.2 ± 1.9	Light to moderate
REMOTE	3.7 ± 0.5	4.4 ± 1.8	3.7 ± 1.9	3.4 ± 2	3.3 ± 1.9	Light to moderate
SHAM	0.2 ± 0	0.2 ± 0.5	0.2 ± 0.5	0.2 ± 0.5	0.2 ± 0.5	No discomfort


### Maximal Voluntary Contraction Task

No main effect of condition was evident for either peak force (kg), area under the curve (kg) during the 30-s MVC (*P* > 0.05, **Table 3**).

## Discussion

The aim of this study was to examine the exercise-mediated changes in artery diameter and blood flow pattern in response to IPC and REMOTE IPC in healthy individuals. We also examined whether IPC and REMOTE IPC could prevent the usual decline in post-exercise vascular function. The novel findings from the current study are (i) brachial artery diameter was greater during exercise following IPC when compared to REMOTE IPC, but this does not translate to a greater conduit artery blood flow between IPC and REMOTE IPC and (ii) neither IPC nor REMOTE IPC prevented the attenuation in brachial artery FMD following 30 min of hand grip exercise *versus* a SHAM condition. Taken together this data suggest that IPC impacts the vasculature more during exercise when compared to REMOTE IPC, leading to a larger diameter change for a given shear stress. However, neither protocol (IPC or REMOTE IPC) was found to enhance blood flow during exercise or prevent the drop in vascular function after exercise.

### Hemodynamics During Handgrip Exercise

This is the first study to compare the effects of two different IPC protocols (local and REMOTE IPC) on exercise-mediated conduit artery diameter and blood flow. The current data show IPC resulted in a larger increase in conduit artery diameter that was maintained throughout exercise *versus* REMOTE IPC. While we did not perform a diameter measurement immediately prior to exercise, the larger diameter was evident in the first minute during exercise and continued to remain larger throughout exercise. Whether IPC exerted this dilatory response prior to the onset of exercise *versus* REMOTE IPC could not be determined in our design. The capacity of IPC to have direct and immediate effects on vasodilation of the conduit arteries infers that IPC could be a useful tool in improving arterial health. For example, in line with the ability of repeated exercise to enhance vascular function (Thijssen et al., 2010), regular episodes of IPC have similarly been shown to enhance arterial health (Jones et al., 2015). The observations in the present study provide further support for the potential benefits of IPC on vascular health.

Potential explanations for the larger diameter between IPC and REMOTE IPC include different impact of shear stress. Based on our observations, shear stress levels did not increase after IPC during exercise. While not measured in the current study, shear stress may have mediated larger nitric oxide release at the site of ischemia, or adenosine-mediated actions as a direct consequence of local IPC (Tapuria et al., 2008; Joyner and Casey, 2014), contributing to a larger diameter increase observed in the current study. When accounting for differences in mean shear rate during exercise, both IPC and REMOTE IPC resulted in lower blood velocity *versus* SHAM. It could be hypothesized that an ischemia-induced increase in diameter following IPC was responsible for producing lower velocity throughout exercise compared to SHAM; however, REMOTE IPC exerted similar responses in comparison, without significantly lower vasodilation response *versus* IPC. The underlying changes in blood velocity responses in both (REMOTE) IPC conditions, when compared to SHAM, are therefore unclear and may deserve further investigation.

An alternative explanation is that IPC affects the sympathetic nervous system (SNS), given that vascular tone is the product of the competitive balance between intrinsic local vasodilator function and adrenoceptor-mediated vasoconstriction. Possibly, IPC caused a reduction in sympathetic nerve activity (SNA), leading to a relative increase in artery diameter during exercise. Lower SNA has been observed following limb ischemia reperfusion injury preceded by IPC using the gold standard microneurography (Lambert et al., 2016). However, it has recently been reported that no change in SNA was present during static handgrip at 30% MVC, following IPC *versus* a SHAM condition (Incognito et al., 2017). Based on the available evidence, we cannot currently elucidate this mechanism. Given the larger diameter observed in the current study, however, we can state that IPC applied locally may exert a larger influence on the vasculature during exercise.

Despite the changes in diameter, this did not accommodate a larger blood flow during exercise. In fact, we even observed a lower mean and antegrade shear following IPC when compared to REMOTE IPC throughout the exercise. This was especially apparent during the first 20-min of exercise. Our findings are in line with a previous study, which found that REMOTE IPC (lower-limb) prior to handgrip exercise to exhaustion did not alter brachial artery blood flow (Barbosa et al., 2015). Nonetheless, this previous study found enhanced time-to-exhaustion after REMOTE IPC. Possibly, local redistribution of blood and/or changes in oxygen extraction (in the presence of preserved total blood flow to a limb) may explain the ergogenic effects observed in those studies. In support of this hypothesis, some studies have reported enhanced tissue oxygenation as an index of muscle perfusion capacity using NIRS when exercise is preceded by IPC (Paradis-Deschênes et al., 2016). Taken together, exercise-induced blood flow to the exercising limb unlikely increases after (REMOTE) IPC. Future work should explore whether local processes, contributing to redistribution of blood flow or oxygen uptake, may contribute to the potential ergogenic effects of (REMOTE) IPC, as currently it is unclear to an ischemia-induced enhancement in conduit artery vasodilation would serve benefit to exercise performance when applied in whole body performance models.

### Brachial Artery Endothelial Function

Upon cessation of continuous exercise of a sufficient intensity, brachial artery FMD displays a biphasic response characterized by an immediate reduction and a return toward pre-exercise levels 1-h post-exercise (Birk et al., 2013). Previously, Bailey et al. (2012a) demonstrated that a bout of REMOTE IPC was capable of attenuating this immediate post-exercise reduction in FMD after a 5 km-TT on the treadmill. In the current study, neither IPC nor REMOTE IPC prevented the exercise-induced reduction in FMD *versus* SHAM exercise. There are a number of potential explanations for these contrasting findings. Firstly, handgrip exercise activates <1 kg of muscle *versus* larger muscle mass exercise such as running which can activate up to 10–15 kg of muscle (Râdegran et al., 1999; Joyner and Casey, 2014). Even during intense handgrip exercise, only ∼70% of the available delivered oxygen is extracted from arterial blood, suggesting the muscles in these modalities may be over-perfused when working in isolation (Joyner and Casey, 2014). While small muscle mass exercise is necessary to investigate the localized effects of exercise, the hemodynamic response to this exercise mode is independent of both neural and central regulatory changes that occur in whole body exercise (Thijssen et al., 2010). Therefore, differences in responses between small- and large-muscle mass exercise tasks when measuring post-exercise vascular function are plausible (Joyner and Casey, 2014; Atkinson et al., 2015). A second explanation relates to the intensity of exercise, where strenuous, whole body exercise is associated with instantaneous vascular injury, subsequently leading to reductions in vascular function (Birk et al., 2013). The oxidative challenge as previously discussed is likely lessened in small muscle mass exercise (Joyner and Casey, 2014). Therefore, exercise tasks utilizing large muscle mass at high intensities maybe more insightful when examining the ability of (REMOTE) IPC to prevent post-exercise vascular dysfunction.

In the current study, we observed no changes in peak force (kg) or mean force production (AUC) following either REMOTE IPC or IPC *versus* SHAM. While studies (Paradis-Deschênes et al., 2016) that have employed exercise modalities utilizing larger muscle mass with similar shear rate patterns in blood flow (Thijssen et al., 2009) have demonstrated improved maximal isometric knee-extension performance *versus* a SHAM condition. Aiming to establish the reason as to why, in the current study, we found no difference in 30-s MVC performance between conditions would be speculative. Especially when compared to reports that IPC can augment 30-s maximal performances in larger muscle mass tasks (Kraus et al., 2015) remains unclear. As previously discussed, differences between large- and small-muscle mass high intensity performance are plausible.

When aiming to establish if an optimal dose–response to IPC exists, assessing the impact of both IPC and REMOTE IPC treatment to a standardized intervention may be useful. We previously observed that both IPC and REMOTE IPC protocols resulted in the same cycling time trial performance (Cocking et al., 2017). While no change in power output occurred between (REMOTE)IPC conditions, IPC resulted in lower oxygen uptake *versus* REMOTE IPC, indicative of IPC-induced metabolic alterations (Cocking et al., 2017). In the current study, IPC resulted in a greater brachial artery dilation that was maintained throughout the exercise bout, resulting in lower mean shear rate and antegrade shear when compared to REMOTE IPC.

### Limitations

Firstly, although handgrip exercise provides capability to measure muscle blood flow accurately, the limited “active” muscle mass (<1 kg) likely provides a different management of vasodilation responses to exercise stimuli when compared to whole-body work. It may therefore not be possible to apply these results to larger active muscle mass areas when running or cycling for example. Secondly, the participation number of *N* = 18 was likely inadequate to be sure of detecting changes in blood flow. We would also like to state that due to the lack of a pre-exercise ultrasound measure, we cannot confirm whether the enlargement in brachial artery diameter observed was an exercise-induced response or indeed an ischemia-induced response. Additionally, it may have been that the MVC effort performed at the end-point of steady-state exercise altered the post-exercise vascular function response in the current study. Finally, the difficulty of truly blinding IPC interventions through use of a 20-mmHg SHAM condition produces the possibility that an expectation bias may occur.

In summary, we demonstrate for the first time that IPC performed as a strategy prior to exercise causes enhanced conduit artery vasodilation, maintained during exercise, when compared to REMOTE IPC. While these vascular adjustments do not translate into increase blood flow during exercise, the larger diameter mediated by IPC could indicate acute bouts of IPC contribute to improving the health of arteries.

## Ethics Statement

The study was approved by the local Ethics Committee (Anti-Doping Lab Qatar – IRB F2016000128). All participants provided written informed consent in accordance with the Declaration of Helsinki. Physical Activity Readiness Questionnaires were administered to ensure no participant had any cardiovascular or metabolic health issues that would prevent participation.

## Author Contributions

HJ, DT, MW, SC, and NC developed the concept for this research project. SC performed all data collection and analysis. SC, HJ, and DT drafted and finalized the manuscript. MW, DG, NC, HJ, and DT significantly contributed to the drafts toward the final product and critically reviewed the manuscript. All authors (SC, MW, DG, HJ, DT, and NC) provided valuable comments throughout and insights throughout the process contributing to the final version of this manuscript. All authors (SC, MW, DG, HJ, DT, and NC) approved the final version of this manuscript.

## Conflict of Interest Statement

The authors declare that the research was conducted in the absence of any commercial or financial relationships that could be construed as a potential conflict of interest.
